# Impact of Female Reproductive Hormones on Surgically Induced Hemorrhage: A Translational Animal Model

**DOI:** 10.3390/jcm15187263

**Published:** 2026-09-18

**Authors:** Nima Mehrjoh, Lucía De Miguel Gómez, Gennaro Selvaggi, Claes Ohlsson, Mats Hellström, Lars Rasmusson

**Affiliations:** 1 Department of Oral and Maxillofacial Surgery, Sahlgrenska Academy, University of Gothenburg, 413 90 Gothenburg, Sweden; nima.mehrjoh@gu.se; 2 Department of Obstetrics and Gynecology, Sahlgrenska Academy, University of Gothenburg, 405 30 Gothenburg, Sweden; 3 Department of Plastic Surgery, Sahlgrenska Academy, University of Gothenburg, 413 45 Gothenburg, Sweden; 4 Department of Internal Medicine and Clinical Nutrition, Sahlgrenska Academy, University of Gothenburg, 405 30 Gothenburg, Sweden

**Keywords:** cranio-maxillofacial surgery, orthognathic surgery, perioperative blood loss, female reproductive hormone, estradiol

## Abstract

**Objectives**: To establish an animal model for investigating the impact of female reproductive hormones on perioperative blood loss during cranio-maxillofacial surgery. **Methods**: Forty female Sprague-Dawley rats were sequentially allocated into five groups (*n* = 8 each): one sham-operated group (SHAM) receiving vehicle and four ovariectomized groups (OVX) receiving either the vehicle, 17β-estradiol, progesterone, or follicle-stimulating hormone-β. Successful ovariectomy was verified by monitoring body mass and measuring terminal serum hormone concentrations. Following a 13-day hormonal regimen, all subjects underwent a standardized surgical procedure to induce mandibular hemorrhage. Perioperative blood loss was quantified gravimetrically until complete hemostasis. **Results**: In the primary analysis, total blood loss differed among the four OVX groups (*p* = 0.038), with lower blood loss observed in the 17β-estradiol-treated OVX rats (OVX+E) compared to the vehicle-treated OVX controls (*p* = 0.0495), though sensitivity analyses indicated that this finding should be interpreted with caution. **Conclusions**: Within the limitations of this study, 17β-estradiol treatment was associated with lower perioperative blood loss in ovariectomized female rats following surgically induced mandibular hemorrhage. This finding suggests that estradiol exposure may influence perioperative bleeding and highlights the need for confirmation in larger, adequately powered studies to further investigate the potential role of estradiol in the preoperative risk assessment of female patients undergoing cranio-maxillofacial procedures.

## 1. Introduction

Over the past few decades, major advancements have been accomplished within the field of cranio-maxillofacial surgery, driven by a better understanding of facial anatomy and biomechanics that have come with the adoption of 3D imaging, virtual surgical planning, and CAD/CAM technology. These innovations have allowed for enhanced surgical precision and technique, which in combination with improved anesthesia protocols, have made surgeries safer with shortened recovery times and increased patient satisfaction [1].

However, despite these advancements, perioperative blood loss remains a significant concern, impacting both patient safety and recovery trajectories [2,3,4,5]. Hence, effective management of blood loss is crucial to further improve the safety and efficacy of orthognathic procedures.

While various factors such as surgical technique, patient comorbidities, and anesthetic management have been extensively studied with regard to their impact on perioperative blood loss in orthognathic surgery, the influence of sex hormones remains underexplored, warranting additional research to fully understand its significance [6].

The current body of literature examining the effect of gender on perioperative blood loss in orthognathic surgery has yielded conflicting results, with some studies indicating that sex does play a significant role [2,7], while other studies found no such correlation [8]. Even within the subset of studies that recognize a gender influence, the results are incongruent with some reported data indicating males having a higher propensity for extensive bleeding [7], while other data indicated that women exhibit a higher tendency for increased blood loss [2]. This disparity underscores the need for more rigorous, large-scale studies to determine the true impact of gender on perioperative bleeding tendencies.

The influence of female sex steroids on perioperative blood loss is well-recognized, with previous research indicating that these hormones exert a more pronounced effect in certain organs. Consequently, this differential impact has allowed for the classification of organs as either hormone-dependent or non-hormone-dependent within the context of perioperative blood loss [9]. Although there have been studies that explored the correlation between female sex hormone levels and the degree of perioperative blood loss in different organs [10,11], there remains a paucity of scientific data regarding the potential relationship between female sex hormone levels in the blood and the extent to which this may affect perioperative bleeding during orthognathic surgery. This gap in knowledge necessitates further investigation to enhance our understanding of the underlying mechanisms and its potential clinical implications.

Existing experimental hemorrhage models, including tail-cut, liver punch biopsy, liver laceration, and spleen transection models, have been developed primarily to investigate the systemic consequences of traumatic hemorrhage and hemorrhagic shock [12]. Although these models have provided valuable insights into the global physiological responses to blood loss, they do not reproduce a standardized, surgically induced bleeding event within a defined anatomical region. Consequently, they are not well-suited for investigating site-specific, procedure-related bleeding of the type encountered in cranio-maxillofacial surgery. There is therefore a need for a standardized experimental model that can reproduce surgically induced hemorrhage at a defined cranio-maxillofacial site, thereby enabling the biological and procedural determinants of perioperative blood loss, including hormonal status, to be investigated under controlled conditions.

To address this gap, the present study aimed to develop a standardized animal model of surgically induced cranio-maxillofacial hemorrhage and to use this model to investigate the impact of female reproductive hormones on perioperative blood loss. We hypothesized that circulating female reproductive hormones modulate the magnitude of perioperative blood loss in this surgical setting. Establishing such a model may provide an experimental foundation for a broader understanding of the relationship between endocrine status and perioperative bleeding during cranio-maxillofacial interventions, with the longer-term goal being improved preoperative risk stratification, perioperative management and postoperative care for female patients.

## 2. Materials and Methods

### 2.1. Ethics

The study adhered to the European Community Guidelines (Directive 2010/63/EU), ARRIVE guidelines, and 3Rs principles for animal research. Ethical approval was obtained from the Malmö/Lund Animal Experimentation Ethics Committee (Dnr. 5.8.18-17791/2023 and date of approval: 20 December 2023).

### 2.2. Study Design

#### 2.2.1. Animals

Forty female Sprague-Dawley rats (10 weeks old, 282 g ± 13 g; Janvier Laboratories, Le Genest-Saint-Isle, France) housed at the Experimental Biomedicine core facility (Gothenburg, Sweden; 2–3 per cage, temperature: 21 ± 1 °C, relative humidity: 45–70%, 12-h light/dark cycle: 07:00–19:00) with standard laboratory diet and water ad libitum were used.

#### 2.2.2. Grouping and Allocation

Animals were numbered 1–40 and assigned to five groups (group labels A–E; *n* = 8 per group) using a cyclic allocation scheme in a repeating A–E sequence performed by an animal technician at the Experimental Biomedicine Core Facility, with treatment allocation concealed from the research team throughout the experimental procedures. As this method lacks a random component, results should be interpreted with the possibility of allocation bias.

#### 2.2.3. Sample Size

The initial group size was determined based on an earlier comparable rodent hemorrhage model in which six animals were used per group across four rat models of uncontrolled hemorrhage [12]. The group size of eight in the study presented herein was used to provide a margin against procedure-related attrition and were further based on prior experience with rodent studies and also considering feasibility and animal-welfare considerations under the 3Rs framework. No formal a priori power calculation was performed.

#### 2.2.4. Experimental Protocol

**Figure 1:**
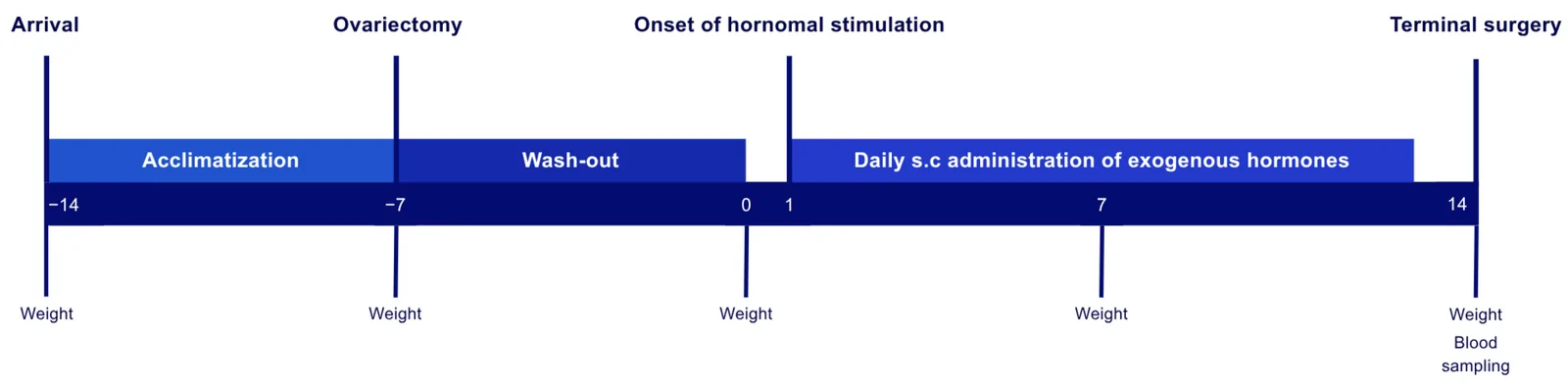
Overview of the research design and experimental process.

Four groups underwent ovariectomy (OVX) and one sham-operation where the ovaries were exposed but not removed (SHAM). Following a seven-day washout period, all subjects received 13 days of daily treatment with the vehicle (SHAM and OVX, respectively), progesterone (OVX+P), 17β-estradiol (OVX+E), or rat follicle-stimulating hormone-β (OVX+FSH). Body mass was monitored as a physiological indicator of successful gonadectomy. Serum hormone concentrations of hormones were measured terminally for further verification of gonadectomy. Post-treatment, all groups underwent a standardized surgical procedure to induce mandibular hemorrhage, allowing for a comparative analysis of perioperative blood loss across groups (Figure 1).

Treatment formulations were prepared and coded by laboratory personnel and administered at matched injection volumes across all groups to help preserve blinding. The research team involved in hormone administration, surgery, blood-loss measurement, and data collection was blinded to treatment identity corresponding to each letter code throughout the study. Group identities were only revealed after the completion of statistical analyses.

Predefined exclusion criteria included (1) death during anesthesia or surgery, (2) adverse events: unexpected organ or tissue injury during surgery, (3) anesthesia-related complications that substantially affected the experimental procedure or outcomes, and (4) severe postoperative infection or wound dehiscence. These criteria were predefined and applied under blinded conditions, prior to unblinding for statistical analysis.

### 2.3. Hormonal Replacement

#### 2.3.1. Ovariectomy

Following acclimatization (7 days), all subjects underwent OVX/SHAM according to the following protocol: induction of anesthesia with 5% isoflurane and maintenance with 2.5%. The surgical site was shaved and disinfected. Subjects were laid prone on a heating pad. Bilateral transverse skin incisions (1 cm) were made caudal to the costal margin and lateral to the spine. The posterior portion of the external oblique muscle was dissected, exposing the adipose tissue, ovaries, and uterus. For OVX, the fallopian tubes were ligated, and both ovaries with periovarian fat tissue were excised. The uterus was then repositioned, muscle tissue sutured, and skin closed with staplers. The SHAM procedure was identical, except that the ovaries were exposed and repositioned without excision. Perioperative analgesia (24 h pre- to 24 h post-op) consisted of buprenorphine (0.05 mg/kg SL q12h, 4 doses; Actavis Inc., Parsippany-Troy Hills, NJ, USA) and meloxicam (1.5 mg/kg SC q24h, 2 doses; Labiana Life Sciences S.A., Barcelona, Spain).

#### 2.3.2. Exogenous Hormone Administration

Post-OVX/SHAM, a 7-day washout period was applied for endogenous hormone clearance. Subsequently, all animals received daily injections (SC, dorsal, 07:00–09:00 a.m., 13 days). OVX animals received either sesame oil (vehicle; Merck Life Science AB, Solna, Sweden), 17β-estradiol (250 μg/kg; LGC Standards GmbH, Wels, Germany), progesterone (14 mg/kg; Merck Life Science AB, Solna, Sweden), or rat follicle-stimulating hormone-β (5 μg/kg; Abbexa Ltd., Cambridge, UK). The SHAM group received sesame oil (vehicle; Merck Life Science AB).

#### 2.3.3. Verification of Ovariectomy

Body mass was monitored, as a successful gonadectomy is indicated by a significant body mass increase. Measurements were taken at five time points: baseline at animal arrival to the facility, day of OVX, day of hormonal stimulation onset, one week post- hormonal stimulation, and day of terminal surgery.

Terminal blood samples of 500 μL were obtained on day 14 through cardiac puncture, 24 h after the final daily hormonal injection. All samples were centrifuged (20 min, 2400 rpm) within 60 min after collection, and the extracted serum was stored at −80 °C. Stored serum samples were analyzed for estradiol and progesterone using high-sensitivity liquid chromatography–tandem mass spectrometry according to a previously published method [13]. The lower limit of quantification (LLOQ) was 1.0 pg/mL for estradiol and 5.6 pg/mL for progesterone. Concentrations below the LLOQ were assigned a value of LLOQ/2 for statistical analysis.

### 2.4. Terminal Surgery

#### 2.4.1. Presurgical Preparations

Anesthesia was induced (SC, mid-dorsal) with a ketamine hydrochloride (75 mg/kg; Intervet International B.v, Boxmeer, The Netherlands) and dexmedetomidine (0.5 mg/kg; Orion Pharma AB, Danderyd, Sweden) mixture (125 µL/100 g body mass). The animal was shaved and positioned in lateral recumbency under a stereotaxic apparatus with limbs secured. A transparent adherent drape isolated the operative field and stabilized the cranium. Core body temperature was maintained (37 °C; homeothermic blanket control unit) to prevent hypothermia-induced alterations.

#### 2.4.2. Surgical Access

**Figure 2:**
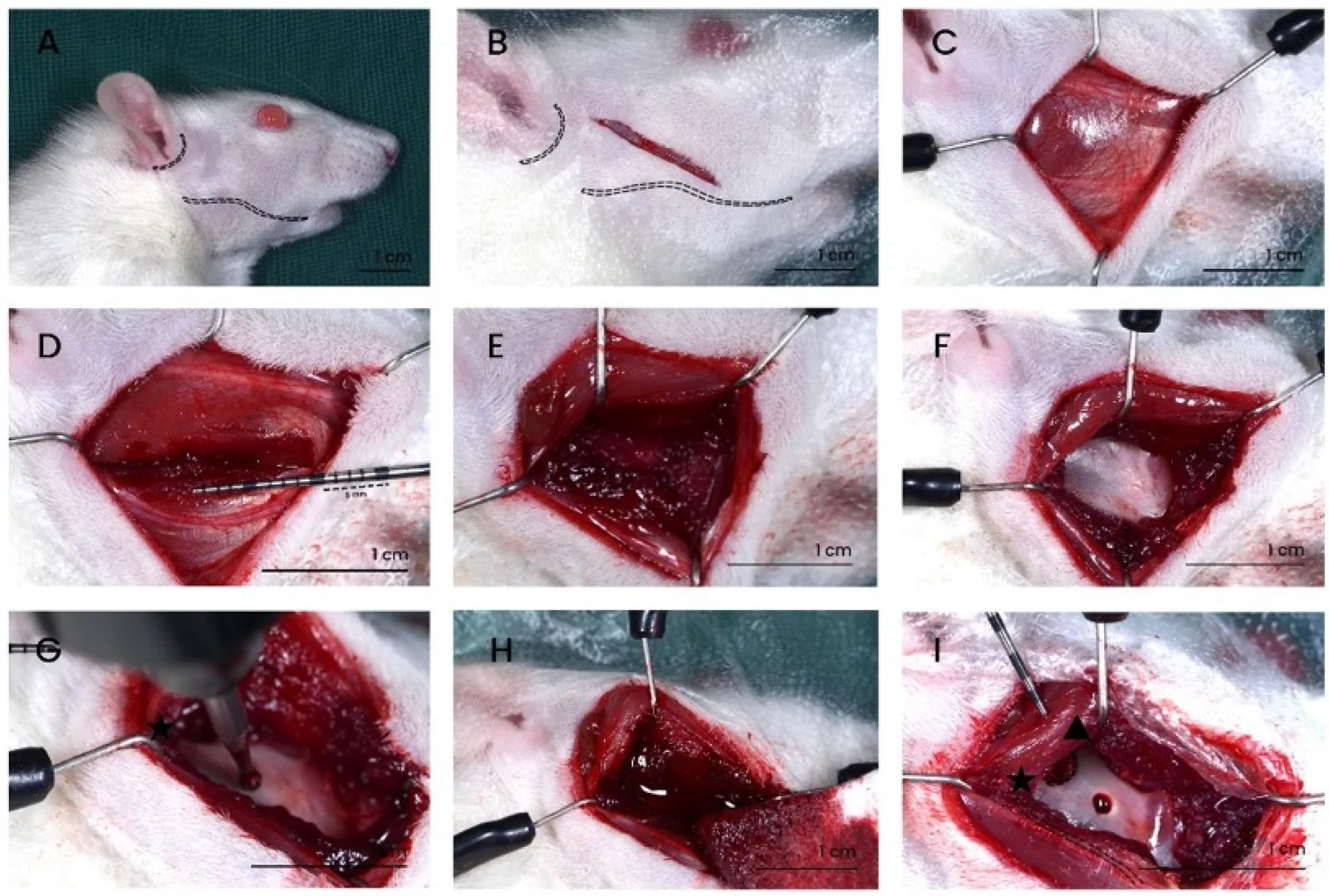
Terminal surgical procedure. (**A**,**B**) Location of the incision site. The inferior mandibular border and anterior inferior tragus margin (dashed lines). (**C**) Exposure of the masticatory musculature. (**D**–**F**) Full-thickness incision, followed by periosteal elevation to access the cortex. (**G**) Creation of the burr hole defect using a stereotaxic frame-guided surgical drill. (**H**) Collection of blood loss using gauze swabs. (**I**) Anatomical landmarks as reference for burr hole localization: condylion (▲), the superior-most and posterior-most point on the mandibular condyle; gonion (★), the inferior-posterior-lateral point at the mandibular angle; and mandibular borders including the posterior ramus and inferior corpus (dashed lines). Scale bar = 1 cm.

An oblique incision (anteroinferior, 1.5 cm) was made from the tragus margin to the inferior mandibular border. Blunt dissection of subcutaneous tissue and superficial muscular fascia was performed to expose the masticatory musculature. Mandibular landmarks were palpated to determine the optimal vertical plane for the subsequent incision, ensuring access to the lateral portion of the mandibular ramus. A full-thickness incision (longitudinal, 2 cm) was made on the horizontal axis of the masseter muscle. Single hook retractors were used for muscle retraction. The buccal periosteum was stripped using a periosteal elevator, exposing the cortical bone (Figure 2).

#### 2.4.3. Induction of Surgical Hemorrhage

A standardized osseous defect was created in the ramus mandibulae using a drill (ISO 016, steel, spherical, Hager & Meisinger GmbH, Neuss, Germany) mounted on a stereotaxic apparatus. Anatomical landmarks for defect location included: inferior mandibular border, condylion, gonion, and posterior border of mandibular ramus. Soft tissue hemostasis was achieved with gauze swabs. The drill was then manually lowered perpendicular to the predetermined location using the stereotaxic apparatus (Harvard Apparatus, Holliston, MA, USA), penetrating the mandibular cortex and medullary cavity, stopping at resistance loss, also indicating penetration of the lingual cortex (Figure 2).

#### 2.4.4. Measurement of Blood Loss

Blood loss was collected using gauze swabs and measured at 15-s intervals following hemorrhage induction until complete hemostasis; the swabs were replaced at each new interval. Gravimetric analysis was employed (blood saturated swab mass − dry swab mass), applying an approximate density-based conversion of 1 g of swabs weight to 1 mL of blood. No irrigation fluid or other fluid was introduced into the surgical field, precluding dilutional errors in the measurement. Upon hemostasis, animals were euthanized under continued anesthesia by cardiac puncture.

### 2.5. Statistical Analyses

Statistical analyses were performed using GraphPad Prism 10.3 and R 4.3.1 (R Core Team, Vienna, Austria). Significance was set at *p* < 0.05. Body mass changes were assessed using two-way repeated-measures ANOVA with Tukey’s test for multiple comparisons, normality was assessed using the Shapiro–Wilk test. Serum hormone concentrations were assessed for normality using the Shapiro–Wilk test. As normality was violated for at least one group for both hormones, non-parametric tests were used: Mann–Whitney tests were used to compare SHAM and OVX animals, and Kruskal–Wallis tests with Dunn’s post hoc test were used to compare among the OVX groups. Total blood loss was analyzed in two separate comparisons: Welch’s *t*-test was used to compare the SHAM and OVX animals, and one-way ANOVA with Dunnett’s post hoc test was used to compare among the OVX groups; normality was assessed using the Shapiro–Wilk test and homoscedasticity using Levene’s test (median-centered/Brown–Forsythe).

## 3. Results

A total of five animals were excluded from the study. Four were excluded due to procedural complications: mortality (*n* = 2, OVX and SHAM groups) and anesthetic overdose prior to terminal surgery (*n* = 2). One additional animal, in the OVX+E group, was excluded per protocol due to an intraoperative bone fracture. The final overall sample size was *n* = 35: SHAM (*n* = 7), OVX (*n* = 6), OVX+P (*n* = 8), OVX+FSH (*n* = 7), and OVX+E (*n* = 7).

### 3.1. Animal Weight Monitoring

**Figure 3:**
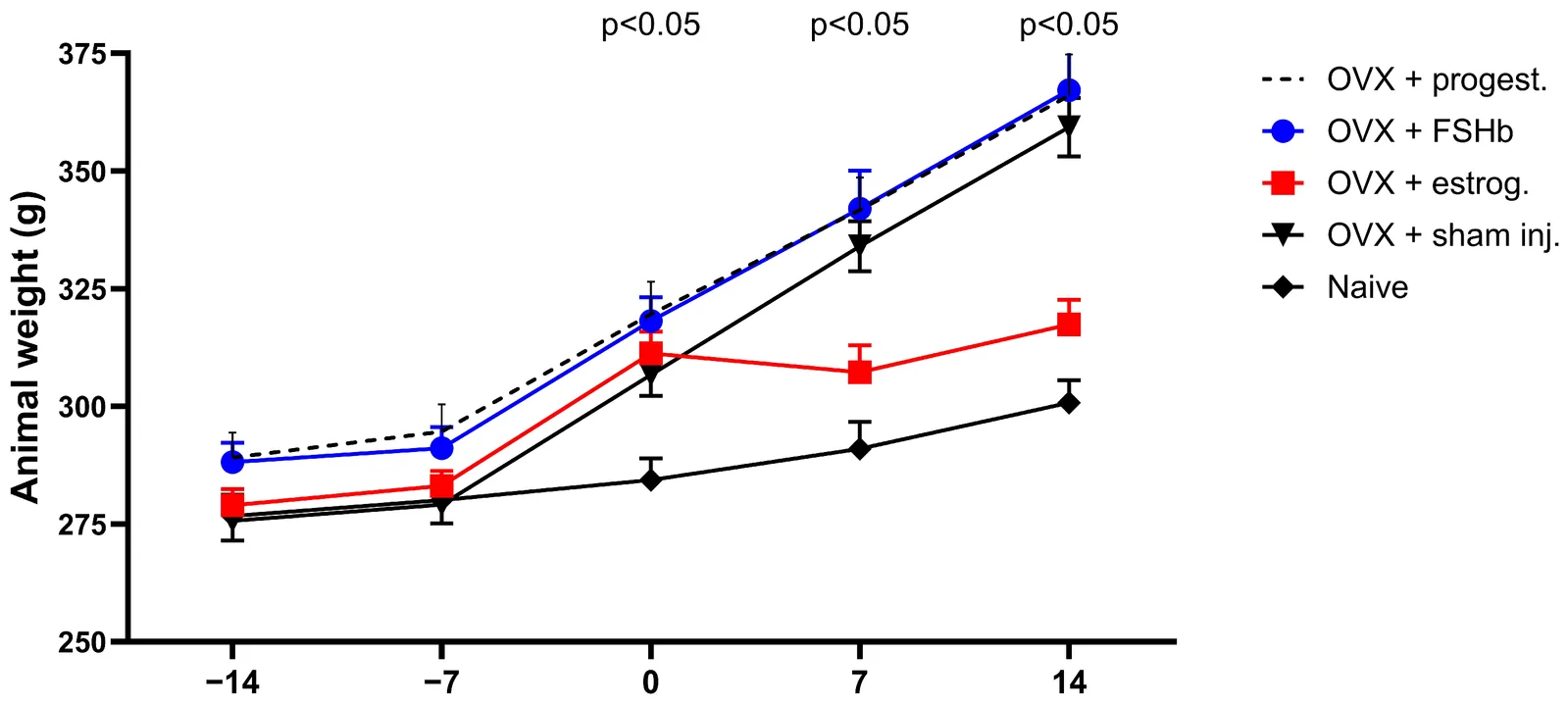
Measurements of animal body mass across five time points. Arrival (day −14), day of ovariectomy (day −7), end of the wash-out period (day 0), 7 days post-hormonal stimulation (day 7), and 14 days post-hormonal stimulation (day 14).

Statistically significant differences were seen between all OVX groups and SHAM at the first weight measurement time point post-OVX (*p* < 0.001, d −7 vs. d0). Significant differences in body mass continued in all OVX groups compared to SHAM after the onset of hormonal stimulation at all other post-OVX measurement time points (*p* < 0.001 for all OVX groups at d0 vs. d7 and d7 vs. d14, except OVX+E). After the onset of hormonal stimulation, OVX+E demonstrated attenuated weight gain compared to other OVX groups, but still a statistically significant increase compared to SHAM (*p* = 0.021 d0 vs. d7; *p* = 0.010 d7 vs. d14) (Figure 3).

### 3.2. Serum Hormone Levels

**Figure 4:**
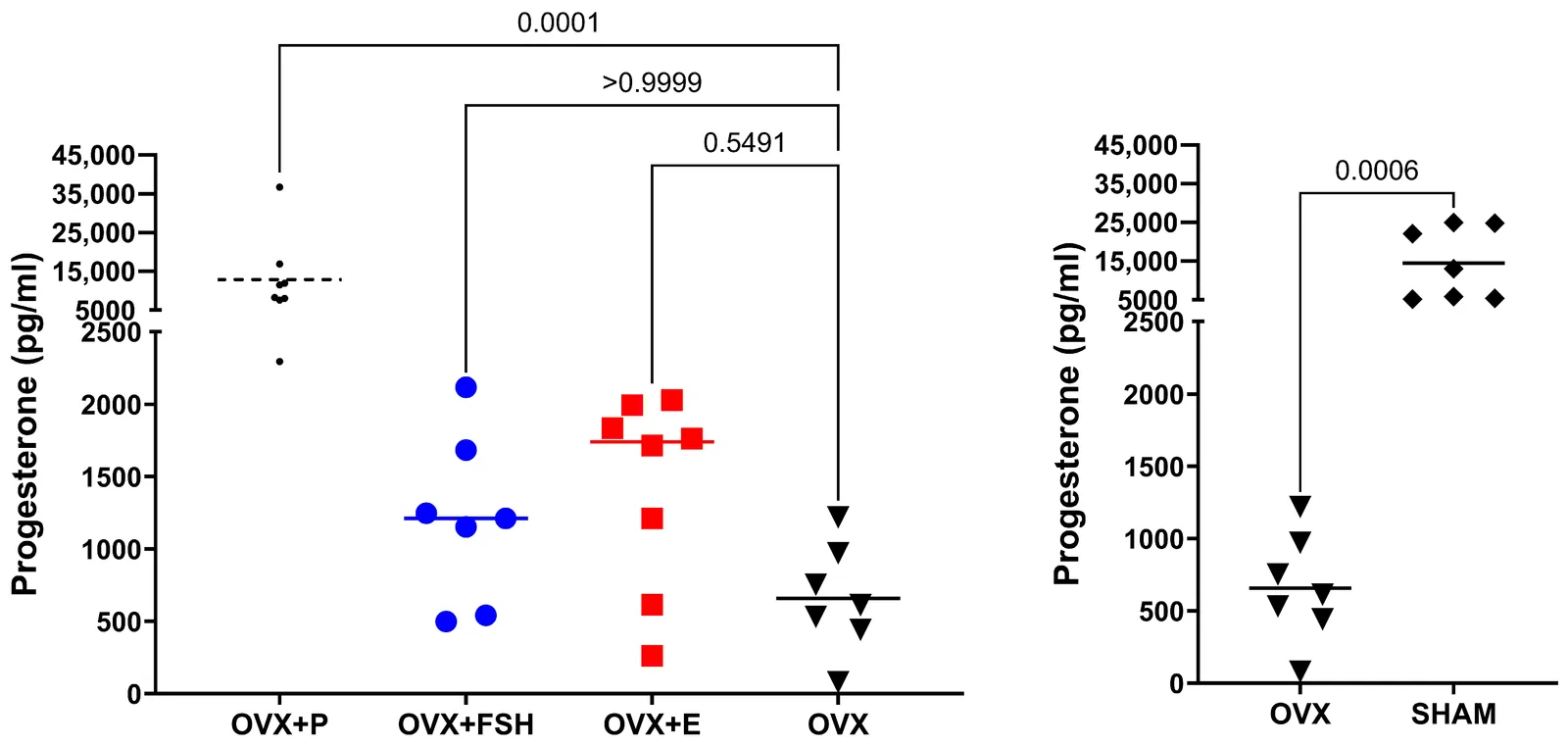
Serum progesterone levels. Terminal (day 14) serum progesterone concentrations per group. (**Left**) Comparison among OVX groups. (**Right**) Comparison between untreated OVX and SHAM animals.

Normality was violated for at least one OVX group for both hormones, and non-parametric tests were therefore used. Serum progesterone concentrations (median [IQR], pg/mL) were SHAM 13,057.0 [5605.0–23,449.0], OVX 612.0 [487.0–862.0], OVX+P 9865.5 [7924.0–13,159.8], OVX+FSH 1211.0 [846.5–1465.5], and OVX+E 1739.0 [1061.8–1875.5]. For progesterone, SHAM animals had significantly higher levels than OVX (Mann–Whitney, *p* < 0.001). Among the OVX groups, a Kruskal–Wallis test revealed a significant overall difference (H = 19.98, *p* < 0.001); Dunn’s post hoc test showed OVX+P was significantly higher than OVX+FSH (*p* = 0.0114), OVX+E (*p* = 0.0437), and OVX (*p* < 0.001), with no differences between OVX+FSH, OVX+E, and OVX (Figure 4).

**Figure 5:**
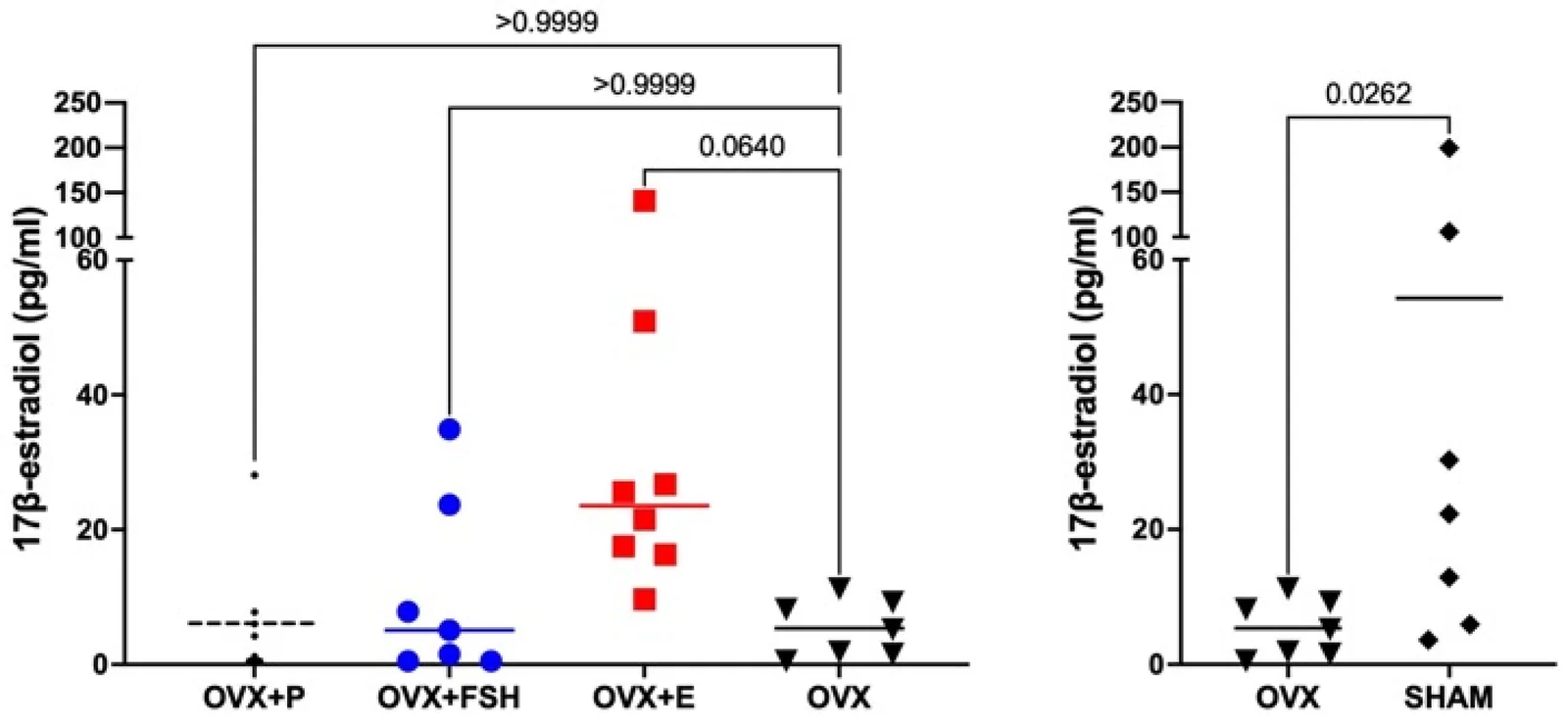
Serum estradiol levels. Terminal serum estradiol (day 14) concentrations per group. (**Left**) Comparison among OVX groups. (**Right**) Comparison between untreated OVX and SHAM animals.

Serum estradiol concentrations (median [IQR], pg/mL) were: SHAM 22.3 [9.4–68.2], OVX 5.2 [1.7–8.7], OVX+P 2.7 [0.5–6.4], OVX+FSH 5.1 [1.0–15.8], and OVX+E 23.6 [17.2–32.8]. For estradiol, SHAM animals also exhibited significantly higher levels than OVX (Mann–Whitney, *p* = 0.026). Among the OVX groups, a Kruskal–Wallis test revealed a significant overall difference (H = 11.69, *p* = 0.0085); OVX+E was significantly higher than OVX+P (*p* = 0.0098), but not significantly different from OVX+FSH (*p* = 0.113) or OVX (*p* = 0.064), with no differences between OVX+P, OVX+FSH, and OVX (Figure 5).

### 3.3. Surgically Induced Blood Loss

Group sizes were as follows: SHAM (*n* = 7), OVX (*n* = 6), OVX+E (*n* = 7), OVX+FSH (*n* = 7), and OVX+P (*n* = 8).

Mean ± SD total blood loss was highest in the OVX group (1409.8 µL ± 1030.6), followed by OVX+FSH (1283.1 µL ± 941.6), SHAM (1225.0 µL ± 1297.9), OVX+P (582.1 µL ± 397.9), and OVX+E (416.4 µL ± 293.0).

**Figure 6:**
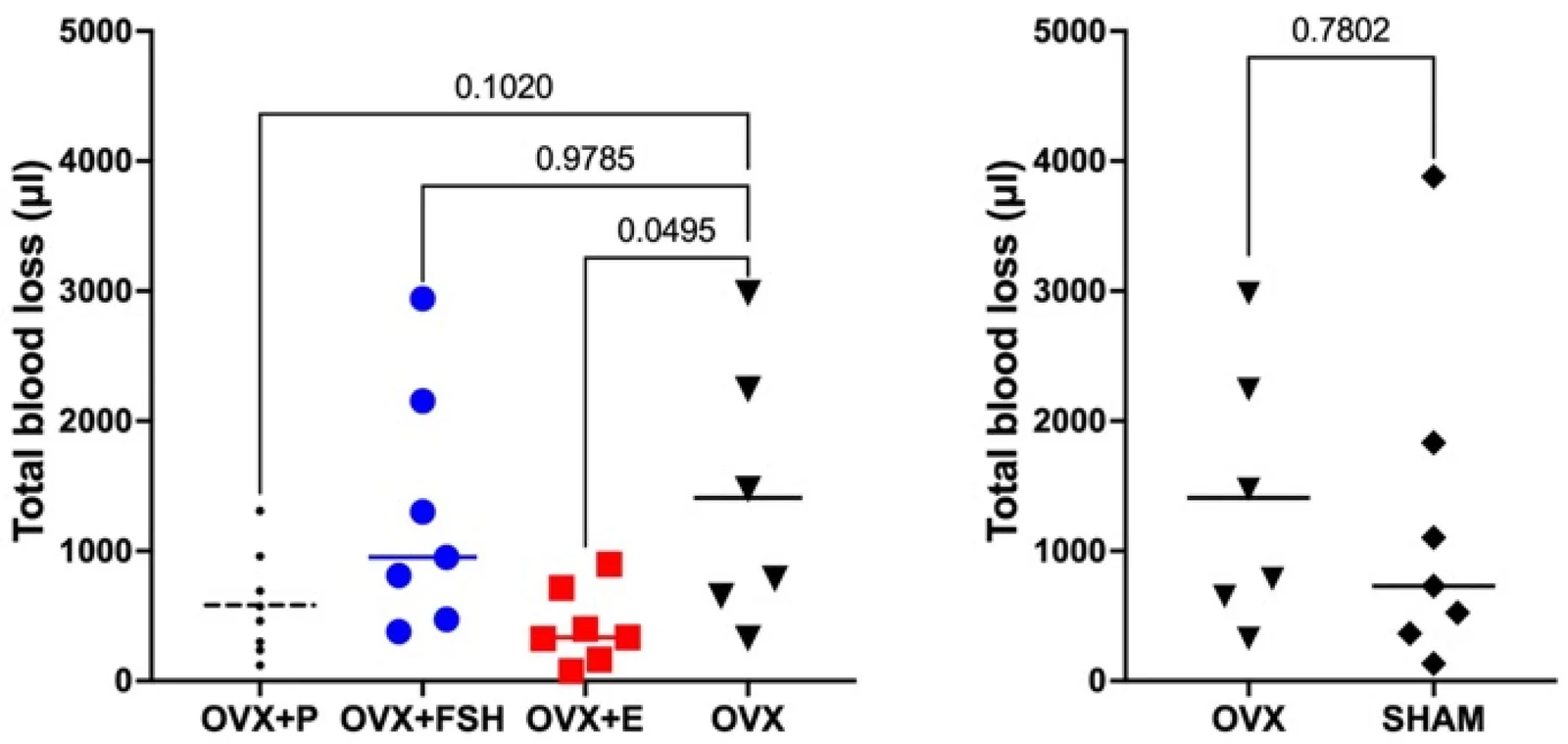
Total perioperative blood loss. Total perioperative blood loss per group, based on the primary analysis (excluding the OVX+E animal with a procedural bone fracture). (**Left**) Comparison among OVX groups. (**Right**) Comparison between untreated OVX and SHAM animals.

Welch’s *t*-test revealed no significant difference in total blood loss between SHAM and OVX (*p* = 0.780). Levene’s test (median-centered/Brown–Forsythe) did not indicate a significant difference in variances among OVX-groups (F(3, 24) = 2.62, *p* = 0.074). One-way ANOVA among OVX groups revealed a significant overall difference (*p* = 0.038). Post hoc Dunnett’s test indicated a significant reduction in total blood loss in OVX+E compared to the OVX controls (mean difference = 993.4 µL, 95% CI: 1.7–1985.1 µL; Hedges’ g = 1.27, *p* = 0.0495), while no significant differences were observed for OVX+P versus OVX (*p* = 0.1020) or OVX+FSH versus OVX (*p* = 0.9785) (Figure 6).

Two additional sensitivity analyses were performed. First, to assess robustness to the unequal group variances, a Brown–Forsythe ANOVA and Welch ANOVA with Dunnett’s T3 post hoc test was performed among the OVX groups. The omnibus test was not significant under either variance-robust approach (Brown–Forsythe ANOVA F = 3.01, *p* = 0.068; Welch F = 2.98, *p* = 0.075), and the OVX+E versus OVX comparison did not reach statistical significance under this method (mean difference = 993.4 µL, 95% CI: −395.6 to 2382.4 µL, *p* = 0.159). Second, to assess robustness to the exclusion of the OVX+E animal with a procedural bone fracture, the one-way ANOVA and Dunnett’s post hoc comparison among the OVX groups was repeated with this animal’s data reincluded (OVX+E *n* = 8, mean ± SD: 617.1 ± 629.2 µL). The omnibus ANOVA was no longer significant (F = 2.30, *p* = 0.102), and the OVX+E versus OVX comparison did not reach statistical significance (mean difference = 792.7 µL, 95% CI: −230.3 to 1815.7 µL; Hedges’ g = 0.90; *p* = 0.152).

## 4. Discussion

### 4.1. A Novel Cranio-Maxillofacial Hemorrhage Model

To our knowledge, this study establishes the first standardized animal model of perioperative hemorrhage in the cranio-maxillofacial region. Existing experimental hemorrhage models, including tail-cut, liver punch biopsy, liver laceration and spleen transection models, have primarily been developed to investigate the systemic consequences of traumatic hemorrhage and hemorrhagic shock [13]. In contrast, the present model was specifically designed to reproduce surgically induced bleeding in a defined cranio-maxillofacial anatomical region. The model therefore addresses an important gap in the experimental literature and may serve as a platform for future studies investigating biological, pharmacological, and procedural factors that influence perioperative blood loss.

Successful ovariectomy was confirmed by two independent measures. First, all OVX groups demonstrated significantly greater increases in body mass compared with SHAM animals from the first post-OVX assessment onward, consistent with the established metabolic consequences of ovarian hormone withdrawal [14]. Notably, OVX+E animals exhibited attenuated weight gain compared with the untreated OVX animals, consistent with the known effects of estradiol on body mass regulation [14], although weight gain remained significantly greater than in the SHAM animals. Second, terminal serum hormone analyses confirmed the successful induction of ovarian hormone deficiency, with OVX animals exhibiting significantly lower progesterone and estradiol concentrations than SHAM animals. As a post hoc assessment, serum hormone concentrations were additionally evaluated to assess the effect of hormone administration. Progesterone concentrations were significantly increased in OVX+P animals compared with the untreated OVX animals. However, the serum estradiol concentrations in OVX+E animals were higher than in the untreated OVX animals, although this difference did not reach statistical significance. This may partly reflect the pharmacokinetic profile of 17β-estradiol, which undergoes rapid decline in circulating concentrations after administration in rats [15]. As serum samples were collected 24 h after the final injection, the estradiol concentrations may have decreased substantially from the post-injection peak levels. The attenuated body mass gain observed in OVX+E animals provides supportive evidence of biological activity, although it does not directly confirm statistically significant elevated circulating estradiol concentrations at the time of terminal sampling. A small number of animals in the non-estradiol-treated groups exhibited terminal serum estradiol concentrations above 20 pg/mL (OVX+P, *n* = 1; OVX+FSH, *n* = 2), values that are atypical for ovariectomized rats not receiving estradiol. These elevated values may reflect a retained ovarian remnant following ovariectomy in individual animals, or contamination during blood sampling or hormone analysis, and their precise cause could not be determined retrospectively. As non-parametric statistical tests were used for these comparisons, individual extreme values have limited influence on group-level significance, and OVX+P and OVX+FSH remained statistically indistinguishable from OVX despite these values. Collectively, these findings support the successful induction of ovarian hormone deficiency and provide evidence of biological activity following estradiol administration. Nevertheless, the absence of a statistically confirmed elevation in terminal estradiol concentrations should be considered when interpreting the estradiol treatment effect on perioperative blood loss.

### 4.2. Lower Blood Loss Following 17β-Estradiol Treatment

The present study showed a reduction in blood loss in OVX animals receiving 17β-estradiol compared to the untreated OVX controls. This finding was sensitive to the specific analytical approach and should be interpreted with caution. This finding is consistent with clinical data from orthognathic surgery reporting lower blood loss in females compared to male patients [16,17]. Schwaiger et al. further identified gender-specific differences in the anticoagulant system as a key contributing factor to this disparity [7].

Experimental evidence from rodent models supports a hemostatic role for 17β-estradiol, although it appears to be context-dependent. Estradiol has been reported to shorten blood-clotting time in rats [18], which is consistent with the direction of the present findings. However, other studies have reported contrasting effects. For example, chronic estradiol treatment in OVX mice has been associated with prolonged tail-bleeding time and reduced platelet aggregation compared to untreated OVX controls [19]. These apparently conflicting findings may reflect differences between the hemostatic environments of peripheral soft-tissue vessels and osseous surgical sites. In the rat mandible, ovariectomy has been shown to increase intracortical remodeling activity by approximately sixfold [20]. Increased bone turnover has, in turn, been associated with greater intraosseous vascularity [21], which may increase bleeding during bone surgery. Estradiol replacement may therefore reduce surgical hemorrhage partly by attenuating ovariectomy-induced bone remodeling and the accompanying increase in intraosseous vascularity. However, this mechanism was not directly investigated in the present study and remains to be confirmed.

The present findings suggest that estradiol exposure may represent one biological contributor to the previously reported sex-based differences in perioperative blood loss between male and female patients, given that estrogen levels are known to differ substantially between the sexes. Hormonal status, and specifically estradiol, may therefore represent a more biologically relevant determinant of perioperative bleeding than biological sex as a categorical variable alone.

However, these findings are derived from an animal model and cannot be directly extrapolated to clinical populations. They should therefore be interpreted as preliminary evidence suggesting that hormonal status may influence perioperative blood loss. In particular, clinical conditions associated with altered estradiol levels, such as ovarian hyperactivity and exogenous hormone use, may warrant further investigation as potential determinants of perioperative bleeding.

### 4.3. Ovarian Hormone Depletion

No statistically significant difference in blood loss was observed between the SHAM and OVX groups. This suggests that withdrawal of endogenous ovarian hormones did not produce a detectable change in perioperative hemorrhage within the timeframe examined.

Although ovariectomy initially alters hemostatic parameters in rats, some coagulation markers such as thrombin time and fibrinogen concentrations have been reported to return toward levels comparable with sham-operated animals within days to weeks after the procedure [22]. Conversely, vascular changes may develop more gradually. In menopausal rat models, endothelial dysfunction reaches its maximum approximately 12 weeks after ovariectomy [23]. At three weeks post-ovariectomy, systemic hemostatic parameters may therefore already have normalized, while vascular and skeletal changes may not yet have progressed sufficiently to alter surgical blood loss. The absence of a difference between SHAM and OVX animals may thus be attributable, at least partly, to the duration of ovarian hormone depletion in this study.

An additional consideration is that circulating estradiol concentrations fluctuate substantially across the rat estrous cycle. The SHAM animals were not synchronized according to the estrous-cycle phase before terminal surgery. Consequently, hormonal status at the time of surgery may have varied considerably within the SHAM group. This intra-group variability may have reduced the ability to detect differences in blood loss between the SHAM and OVX animals.

### 4.4. FSH and Progesterone Supplementation

FSH replacement had no detectable effect on blood loss. This finding is consistent with the predominantly gonadotropic role of FSH and suggests that the elevated FSH concentrations characteristic of the postmenopausal state does not independently influence surgical bleeding in this model.

Progesterone replacement was associated with a non-significant trend toward reduced blood loss compared with the untreated OVX controls. Although the progesterone effect was not statistically significant, a potential vascular effect of progesterone is biologically plausible. In ovariectomized rats, progesterone treatment has been shown to preserve endothelium-dependent vasodilatation and reduce oxidative stress through modulation of the nitric oxide pathway [24]. However, the present findings do not establish an independent hemostatic effect of progesterone. The observed trend may reflect a true biological effect, insufficient statistical power, or random variation. Further studies with larger sample sizes are required to determine whether progesterone meaningfully influences perioperative hemorrhage in the cranio-maxillofacial region.

### 4.5. Study Limitations

Several limitations should be considered when interpreting the present findings.

Firstly, the terminal nature of the model may elicit compensatory physiological responses associated with substantial acute blood loss, including sympathoadrenal activation and peripheral vasoconstriction [12]. These responses may influence bleeding dynamics and are not fully representative of elective surgery, in which hemorrhage is generally controlled throughout the procedure.

Secondly, the model captures a single acute hemorrhagic event rather than the cumulative perioperative blood loss encountered in clinical surgical procedures. As demonstrated by Schwaiger et al., blood loss following orthognathic surgery is not confined to the intraoperative period. Postoperative bleeding during the first 48 h may contribute substantially to total perioperative blood loss [25]. The present model therefore represents acute surgical bleeding rather than the complete perioperative hemorrhagic course.

Thirdly, the 13-day hormone-treatment period, although sufficient to induce measurable physiological effects, as demonstrated by body-weight trajectories, may not fully reproduce the chronic hormonal phenotypes encountered in clinical populations. Longer treatment and hormone-depletion periods may be required to model the long-term vascular, skeletal, and hemostatic consequences of menopause or hormone replacement therapy.

Finally, the OVX+E versus OVX comparison was based on relatively small and unequal final group sizes (*n* = 6–8). Additional sensitivity analyses, assessing robustness to unequal group variances and to the exclusion of the animal with a procedural bone fracture, suggested that this comparison is sensitive to the specific analytical approach used. Given the small sample size and this sensitivity, the principal finding should be regarded as exploratory rather than confirmatory.

## 5. Conclusions

The present study provides preliminary experimental evidence that estradiol is associated with lower surgically induced hemorrhage in the cranio-maxillofacial region in the used animal model. These findings raise the possibility that perioperative bleeding in this anatomical region could, at least partly, be influenced by hormonal status, with estradiol potentially exerting a modulatory effect on blood loss. If similar associations are confirmed in more adequately powered experimental studies and future clinical studies, estradiol status may warrant consideration as a potential factor in preoperative risk stratification and perioperative management in female patients undergoing surgery. These findings therefore provide a biological rationale and experimental foundation for further investigation, including studies in human cohorts, to explore whether the possible effect observed in this animal model translates to clinically meaningful differences in perioperative blood loss in females undergoing cranio-maxillofacial surgery.

## Data Availability

The original contributions presented in this study are included in the article. Further inquiries can be directed to the corresponding author.
